# p38^MAPK^/MK2 signaling stimulates host cells autophagy pathways to restrict *Salmonella* infection

**DOI:** 10.3389/fimmu.2023.1245443

**Published:** 2023-09-12

**Authors:** Abdulhadi Suwandi, Manoj B. Menon, Alexey Kotlyarov, Guntram A. Grassl, Matthias Gaestel

**Affiliations:** ^1^ Institute of Cell Biochemistry, Hannover Medical School, Hannover, Germany; ^2^ Kusuma School of Biological Sciences, Indian Institute of Technology Delhi, New Delhi, India; ^3^ Institute of Medical Microbiology and Hospital Epidemiology, Hannover Medical School, Hannover, Germany; ^4^ German Center for Infection Research (DZIF), Partner Site Hannover-Braunschweig, Hannover, Germany

**Keywords:** p38^MAPK^, MK2, cell signaling, *Salmonella*, infection, autophagy, xenophagy, MEFs

## Abstract

Autophagy plays an important role in recognizing and protecting cells from invading intracellular pathogens such as *Salmonella*. In this work, we investigated the role of p38^MAPK^/MK2 in modulating the host cell susceptibility to *Salmonella* infection. Inhibition of p38^MAPK^ or MK2 led to a significant increase of bacterial counts in *Salmonella* infected mouse embryonic fibroblasts (MEFs), as well as in MK2-deficient (*Mk2^-/-^*) cells. Furthermore, western blot analysis showed that *Mk2^-/-^* cells have lower level of LC3 lipidation, which is the indicator of general autophagy compared to *Mk2*-rescued cells. In *Mk2^-/-^* cells, we also observed lower activated TANK-binding kinase-1 phosphorylation on Ser172 and p62/SQTM1-Ser403 phosphorylation, which are important to promote the translocation of p62 to ubiquitinated microbes and required for efficient autophagy of bacteria. Furthermore, immunofluorescence analysis revealed reduced colocalization of *Salmonella* with LC3 and p62 in MEFs. Inhibition of autophagy with bafilomycin A1 showed increased bacterial counts in treated cells compared to control cell. Overall, these results indicate that p38^MAPK^/MK2-mediated protein phosphorylation modulates the host cell susceptibility to *Salmonella* infection by affecting the autophagy pathways.

## 1 Introduction

Autophagy is a highly conserved and tightly regulated process of intracellular protein degradation in eukaryotic cells that is activated by cellular stressors such as nutrient starvation, damaged organelles, misfolded proteins and proteotoxic aggregates, infections etc. (1, 2). In this mode of degradation, the cytoplasmic substrates are engulfed into double-membrane vesicles, called autophagosomes, which then fuse with lysosomes for eventual degradation. Autophagy is not only important in cell homeostasis, but is also crucial for the host to defend against intracellular pathogens (3). Selective autophagy, called xenophagy, represents a direct antimicrobial mechanism. It is mediated by ubiquitination and colocalization of pathogens with autophagy receptor proteins such as sequestosome 1(SQSTM1)/p62, nuclear dot protein 52 (NDP52/CALCOCO2) and optineurin (OPTN) (4, 5). Several studies have demonstrated diverse autophagy-evasion mechanisms by intracellular pathogens ranging from blocking of targeting to the autophagosomes, interfering with autophagosome-lysosome fusion to establish their niches inside the cells (5).

*Salmonella enterica* serovar Typhimurium (*S.* Typhimurium) is a Gram-negative and facultative intracellular bacterial pathogens that causes a variety of diseases from gastroenteritis to typhoid fever in humans and mice (6). *S.* Typhimurium uses its needle-like protein complex called type III secretion systems (T3SSs) encoded by *Salmonella* pathogenicity island 1 (SPI-1) and SPI-2 to deliver bacterial effectors to the host cells to manipulate host cell signaling, cytoskeletal and vesicular pathways (7). SPI-1 genes are important for bacterial invasion, whereas SPI-2 genes are required for intracellular bacterial survival (8). Upon invasion into mammalian cells, most *Salmonella* remain in a single membrane-bound compartment called the *Salmonella*-containing vacuole (SCV) that acquires late endosomal markers such as the lysosomal-associated membrane protein 1 (LAMP-1). However, some bacteria escape from the SCV and replicate rapidly in the host cell cytosol. A previous study showed that autophagy targets *Salmonella* found in damaged SCVs and in the cytosol for degradation and restricts *Salmonella* replication (9).

The p38^MAPK^ pathway and its downstream signaling MAPKAP kinase 2 (MK2) are important for the induction of innate immunity and the regulation of inflammatory responses. They are involved in transcriptional and post-transcriptional regulation of cytokine expression (10–12). Our previous studies have shown that MK2-and MK2/3-deficient mice display an impaired inflammatory response against LPS-induced endotoxic shock and increased susceptibility to *Listeria monocytogenes* infection because of impaired production of TNF (13–15). Oral administration of *S.* Typhimurium activated the p38^MAPK^ pathway in mice and inhibition of the p38^MAPK^ pathway using SB203580, a specific reversible p38^MAPK^ inhibitor, was shown to abrogate *S.* Typhimurium-triggered expression of the antimicrobial peptide cryptdin in Paneth cells *in vivo* (16). The cellular and molecular mechanism how p38^MAPK^/MK2 influence the survival of *S.* Typhimurium remains elusive. Here, we demonstrate that the p38^MAPK^/MK2 signaling axis regulates intracellular *S.* Typhimurium survival by modulating autophagy pathways in the host cells.

## 2 Materials and methods

### 2.1 Bacteria

*S.* Typhimurium SL1344 (S. Tm) WT (17), *S.* Tm Δ*invA*, *S.* Tm Δ*sopB*, *S.* Tm Δ*sopE/E2*, *S.* Tm Δ*avrA* and *S*.Tm eGFP (pFPV25.1) (18) were grown overnight at 37°C with shaking in lysogeny broth (LB) supplemented with 100 μg/ml streptomycin, 100 μg/mL ampicillin, or 50 μg/mL kanamycin, when appropriate.

### 2.2 Cell lines

Mouse embryonic fibroblast (MEFs) WT, MK2 deficient cells transduced with retroviral constructs pMMP–IRES–GFP (vector), MK2 deficient cells transduced with retroviral constructs pMMP–MK2–IRES–GFP (MK2), MK2 deficient cells transduced with retroviral constructs pMMP–MK2^KR^–IRES–GFP (MK2-K79R), kinase-dead MK2 mutants in which lysine79 in catalytic subdomain I is replaced with arginine, MK2 deficient HEK293T cells (MK2^-/-^), MK2 sufficient HEK293T (MK2^+/+^), immortalized bone marrow derived macrophages (iBMDM) from MK2 deficient cells transduced with retroviral constructs pMMP–IRES–GFP (vector), MK2 deficient cells transduced with retroviral constructs pMMP–MK2–IRES–GFP (MK2). The generation of MEF cell line was done by cotransfection of primary MEFs with pSV40Tag encoding simian virus 40 large T-antigen and pREP8 plasmid (Invitrogen) in a 10:1 mixture. Colonies were selected with 3 mM histidinol (Sigma) (19). Immortalized murine macrophages (iBMDMs) were established from bone marrow-derived macrophages (BMDM) that were isolated from mice deficient for MK2 or from wild type mice by retroviral infection with a virus encoding both the *v-Raf* and the *v-Myc* oncogenes as described in detail previously (20). Bone marrow derived macrophages (BMDM) were isolated from *Mk2^+/+^* and *Mk2^-/-^* mice were cultivated as described (21). All cells were cultured in DMEM GlutaMAX (DMEM-Life Technologies) supplemented with 10% (v/v) fetal bovine serum and 1x Penicillin Streptomycin.

### 2.3 Gentamicin protection assay

Cells as indicated were seeded in 24-well plates (10^5^ cells/well) [for bacterial count and immunofluorescence analysis (IF)] or 6- well plates (4x10^5^ cells/well) [for western blot (WB)] and incubated overnight in 5% CO_2_ at 37°C. The next day, culture medium was replaced with DMEM without Penicillin Streptomycin and indicated substance such as DMSO, the pan-p38 inhibitor BIRB-796 (BIRB, 1µM), the MK2 inhibitor PF-3644022 (PF22, 5µM), the autophagy inhibitor Bafilomycin A1 (BafA1, 20nM), the mTOR inhibitor Rapamycin (1µM), the TBK1 inhibitor BX795 (1µM), or TNF-α (20ng/mL) for at least one hour before infection. Cells were then infected with different *Salmonella* strains at MOI 10, 100, 500, 1000 (as indicated) for 1 hours. For IF and WB, cells were infected at MOI 100. Cells were washed 3 times with PBS and culture medium was replaced with medium supplemented with 100 μg/mL gentamicin (Sigma) and indicated substances as above for 1 hours to kill extracellular bacteria. For 2 hours post infection time point, cells were lysed in PBS containing 1% (v/v) Triton X-100 and 0.1% (v/v) sodium dodecyl sulfate (SDS) (for bacterial counts) or were fixed with 4% PFA (for IF) or lysed in with Laemmli loading buffer (for WB). The cell lysates were then serially diluted in PBS and plated on LB agar for colony-forming unit (CFU) count. For later time points, culture medium was replaced with a medium supplemented with 10 μg/mL gentamicin (Sigma).

### 2.4 Immunofluorescence

Fixed cells were washed 3 times with PBS, and permeabilized with Triton-X100 (0.25%) in PBS for 30 min at RT. Blocking was done using 4% bovine serum albumin (BSA) in PBS (blocking buffer) for 1 h at 4°C. Cells were then incubated with first antibodies in blocking buffer and incubated overnight at 4°C. The following first antibodies were used: LC3 (mbl international), p62 (Abcam), *Salmonella* O Antiserum Group B (BD Difco), *Salmonella* Typhimurium (Clone: B2363M, Meridian Life Science), LAMP-1 (eBioscience) and Anti-Ubiquitinylated proteins Antibody, clone FK2 (Sigma). After 3 times washing with PBS, secondary antibodies were added and incubated for 1h at RT. The following secondary antibodies were used: Alexa (Ax) 488- conjugated goat anti-mouse IgG, Ax488-conjugated goat anti-rabbit IgG, Ax555-conjugated goat anti-rabbit IgG, Ax568-conjugated goat anti-mouse IgG, Ax568-conjugated goat anti-rat IgG (Thermofisher Scientific). Phalloidin-iFluor 647 (1:1000) (Abcam) and DAPI (Invitrogen) were applied to visualize F-actin and nuclei, respectively. Images were obtained on a Cytation 1 (BioTek) at 20x magnification in 5-10 fields of view per samples using Gen 11 software (BioTek). Brightness and contrast were adjusted using ImageJ 1.53k software. Gen 11 software (BioTek) was used to quantify median fluorescence intensity (MFI) colocalization of LC3 and p62 with S.Tm eGFP and percentage of colocalization of LAMP-1 and ubiquitinated protein with *Salmonella*. GFP staining with size of 0.5 – 5 µm was defined as primary mask to identify the bacteria, then the mask was expanded for 1 µm. For p62 and LC3 colocalization with *Salmonella*, MFI of Ax555 (LC3) and Ax568 (p62) inside the primary mask were measured. The results are defined as the median of Ax555 or Ax568 fluorescence intensity colocalized with STm-GFP. For LAMP-1 and ubiquitin colocalization with *Salmonella*, MFI of Ax568 inside the primary mask were measure. The threshold was determined from highest MFI of the bacteria completely absence of LAMP-1 or ubiquitin staining. Furthermore, the percentage of LAMP-1 or ubiquitin positive staining were calculated and determined. The results are defined as percentage of LAMP-1 or ubiquitin positive staining to total bacteria.

### 2.5 Western blot

Cells were directly lysed by boiling in 2x SDS-PAGE loading buffer then separated by SDS-PAGE and transferred to polyvinylidene difluoride (PVDF) membrane. After blocking with 3% milk in PBS with 0.1% Tween 20, membranes were incubated with primary antibody in PBS with 3%BSA and 0.1% Tween 20. The following primary antibodies were used: LC3 (MBL International), p62 (Abcam), p-p62 Ser403 (MBL International), TBK1/NAK, p-TBK1/NAK Ser172, Atg5 (D5F5U), p-MK2 (Phospho-MAPKAPK-2) Thr222, MK2 (MAPKAPK-2) (Cell Signaling Technology), EF-2 (Santa Cruz Biotech), GAPDH (Abcam). Immunoreactive bands were visualized using appropriate secondary antibodies and enhanced chemiluminescence detection reagents. Images were obtained using the ImageQuant LAS 3000 system.

### 2.6 Statistical analysis

Data were analyzed using Prism V7.0d software (GraphPad). Statistical analysis was done using one-way analysis of variance (ANOVA) followed by Tukey’s multiple comparison test or T-Test as indicated. Graphs display the mean values ± SD, unless stated otherwise.

## 3 Results

### 3.1 p38^MAPK^/MK2 signaling restricts intracellular infection by *Salmonella*

**Figure 1:**
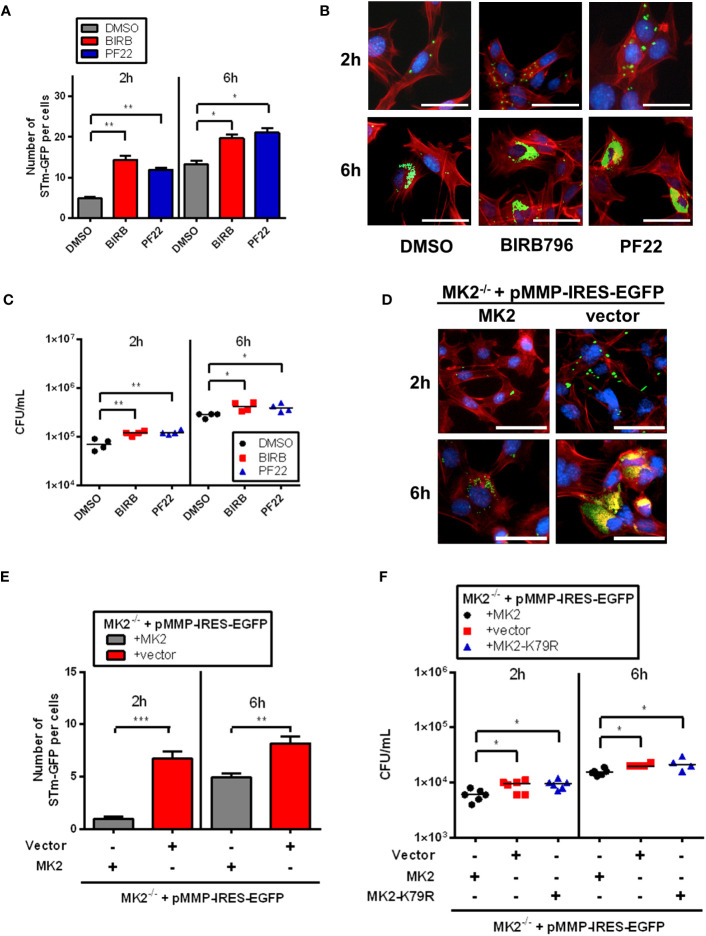
p38MAPK/MK2 signaling influence survival of intracellular *S.* Typhimurium. **(A)** Quantification and **(B)** immunofluorescence staining of MEF WT treated with DMSO (grey), BIRB (red) and PF22 (blue) were infected with STm-GFP (green), fixed at 2h and 6h p.i. Nuclei were stained with DAPI (blue). F-actin (red) were stained with Phalloidin-647. The results are defined as number of STm-GFP per cells. Original magnification: 200x. Scale bars = 100 μm. One-way ANOVA with Tukey’s multiple comparison test, *p<0.05, **p<0.01. **(C)** MEF WT treated with DMSO (black closed circle), BIRB (red closed square) and PF22 (blue closed triangle) were infected with *S.* Typhimurium (STm) WT at MOI 10 for 1 h, washed three times with PBS, then incubated for 1 hours in media supplemented with 100 μg/mL gentamicin. At 2h and 6h p.i, cells were lysed, serially diluted and plated on LB agar plates to quantify intracellular bacteria. The results are defined as colony forming unit (CFU)/mL. One-way ANOVA with Tukey’s multiple comparison test, *p<0.05, **p<0.01. **(D)** Immunofluorescence staining and **(E)** quantification of *Mk2*-rescued (MK2-grey) and *Mk2^-/-^* (red) MEFs were lentivirally transduced with vectors for empty control (vector) were infected with STm-GFP (green), fixed at 2h and 6h p.i. Nuclei were stained with DAPI (blue). F-actin (red) were stained with Phalloidin-647. The results are defined as number of STm-GFP per cells. Original magnification: 200x. Scale bars = 100 μm. Unpaired t-test. **p < 0.01, ***p < 0.001. **(F)** *Mk2*-rescued (black closed circle), *Mk2^-/-^* (red closed square) and MEFs expressing a kinase-dead MK2 mutant in which lysine 79 (*Mk2^K79R^*, blue closed triangle) were infected with *S.* Typhimurium WT at MOI 10 for 1 hour, washed three times with PBS, then incubated for 1 hours in media supplemented with 100 μg/mL gentamicin. At 2h and 6h p.i, the cells were lysed, collected, serially diluted and plated on LB agar plates to quantify intracellular bacteria. The results are defined as CFU/mL. One-way ANOVA with Tukey’s multiple comparison test, *p<0.05. Data points, means and SD of representative results of at least three independent experiments are depicted.

To investigate the role and detailed mechanisms of p38^MAPK^/MK2 pathway in cellular response to *Salmonella* infection, we first performed the gentamicin protection assay for infection efficacy. Therefore, we infected murine embryonic fibroblast (MEFs) with *S*. Typhimurium for 1h, treated cells with gentamicin to inactivate extracellular bacteria, lysed cells and counted bacteria numbers at 2 and 6 hours post infection (p.i.). Interestingly, immunofluorescence analysis of individual cells infected with *S*. Typhimurium expressing GFP showed increased bacterial number after p38^MAPK^ inhibitor BIRB796 (BIRB) and MAPKAPK2 (MK2) inhibitor PF-3644022 (PF22) treatment at 2h p.i. and 6h p.i. (**Figures 1A, B**). Furthermore, we observed increased *S*. Typhimurium counts in the presence of the BIRB and the PF22 (**Figure 1C**). To convincingly prove that MK2 contributes to *S.* Typhimurium control, we performed similar experiments in MK2-deficient MEFs. Consistent with the findings from pharmacological inhibition of p38/MK2 pathway, cells lacking MK2 showed higher intracellular counts of GFP-labelled bacteria compared to cells rescued with retroviral expression of MK2 (**Figures 1D, E**). Furthermore, MK2-deficient MEFs and MEFs expressing a kinase-dead MK2 mutant in which lysine 79 in the ATP-binding catalytic subdomain I is replaced with arginine (19) showed higher bacterial counts compared to wild type *Mk2*-rescued MEFs at 2 hours and 6 hours p.i. (**Figure 1F**). Interestingly, unlike at later time points, at 90 minutes p.i., MK2-deficient and wild-type *Mk2*-rescued MEFs showed similar bacterial counts (**Supplementary Figure 1A**). This indicates that there are similar levels of invasion of *Salmonella* in both cell lines, however the differences arise after cell entry.

Macrophages are important hosts for intracellular *Salmonella* and hence we also verified our findings in primary bone-marrow-derived macrophages (BMDMs). We used immortalized bone marrow-derived macrophages (iBMDM) with a retroviral MK2 rescue and obtained similar results at 4 hours p.i (**Supplementary Figure 1B**). Furthermore, we generated and compared BMDMs from MK2-deficient and wild-type mice in similar assays and observed higher intracellular *Salmonella* counts in MK2-deficient BMDMs (**Supplementary Figure 1C**). Moreover, similar results were observed in MK2-deficient HEK293T cells compared to wild-type control cells (MK2 WT) at 2 hours p.i. (**Supplementary Figure 1D**). Thus, our findings show that p38^MAPK^/MK2-mediated protein phosphorylation modulates the host cell susceptibility to *Salmonella* infection in different relevant cell types.

### 3.2 MK2-deficient MEFs display a higher abundance of cytosolic *Salmonella*

**Figure 2:**
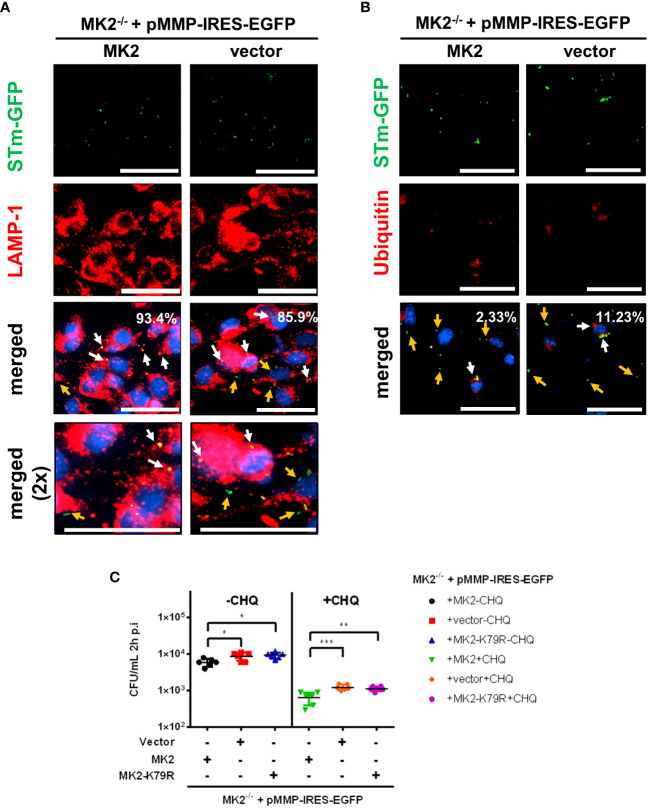
In cells lacking MK2, bacteria are abundance in host cytosol. *Mk2*-rescued (MK2) and *Mk2^-/-^* (vector) were infected with STm-GFP (green) at MOI 100. Infected cells were fixed at 2h p.i, stained with **(A)** anti-LAMP-1 and **(B)** anti-ubiquitin antibody followed by Ax568-labeled secondary antibody (red). Percent colocalization was determined by counting the number of bacteria with higher MFI from predetermined threshold to the total bacteria. White arrows showed colocalized STm, whereas orange arrows showed non-colocalized STm. Nuclei were stained with DAPI (blue). The experiment shown is representative of three independent experiments. **(C)** *Mk2*-rescued (MK2), *Mk2^-/-^* (vector) and *Mk2^K79R^* MEFs were infected with STm WT at MOI 10. Medium with gentamicin (100 μg/mL) and with (+CHQ) or without (-CHQ) 400 μM chloroquine were added 1 hour after infection. At 2h p.i, cells were washed with PBS and lysed. The results are defined as CFU/mL. One-way ANOVA with Tukey’s multiple comparison test, *p<0.05, **p<0.01, ***p < 0.001. The experiment shown is representative of three independent experiments.

After entry into the host cell, *Salmonella* resides within a modified phagosomal compartment termed the *Salmonella*-containing vacuole (SCV) that acquire late endosomal markers, such as LAMP-1. Most intracellular *Salmonella* remain and replicate in the SCV. However, a small percentage of bacteria can damage the SCV and escape into the host cytosol. Cytosolic *Salmonella* are rapidly recognized by the ubiquitin E3 ligases resulting in the formation of ubiquitin chain layer surrounding the bacteria, marked by autophagy adaptor molecules for later downstream autophagy activation to eliminate the bacteria. Meanwhile, *Salmonella* residing in damaged SCVs can also be targeted for autophagic elimination (9). To investigate whether p38^MAPK^/MK2 signaling affects subcellular localization of intracellular bacteria, we infected MEFs with *S*. Typhimurium-GFP and analyzed the samples by co-immunofluorescence microscopy using an anti-LAMP1 antibody. At 2 h p.i., a higher percentage of bacteria colocalized with LAMP-1 (SCV) in MK2 expressing MEFs (93.4%) compared to MK2-deficient MEFs (85.9%) (**Figure 2A**). We also observed a higher percentage of *Salmonella* colocalized with ubiquitin in MK2^-/-^ MEFs (11.23%) suggesting that higher abundance of cytosolic *Salmonella* in *Mk2*-deficient MEFs compared to *Mk2*-rescued MEFs (2.33%) (**Figure 2B**).

Subsequently, we used a combination of chloroquine (CHQ) resistance assay and gentamicin protection assay to quantitatively determine cytosolic *Salmonella* counts. CHQ is a lysosomotropic agent that accumulates only within the SCV and leads to the killing of vacuolar bacteria by a still unknown mechanisms (22). MK2-deficient and MK2^KR^ -rescued MEFs showed higher viable *Salmonella* counts compared to wild-type *Mk2*-rescued MEFs at 2 hours p.i. with or without CHQ treatment (**Figure 2C**), indicating that MEF cells lacking MK2 activity accumulate higher cytosolic *Salmonella* load.

### 3.3 The autophagic capture of *Salmonella* was affected by MK2 in MEFs

**Figure 3:**
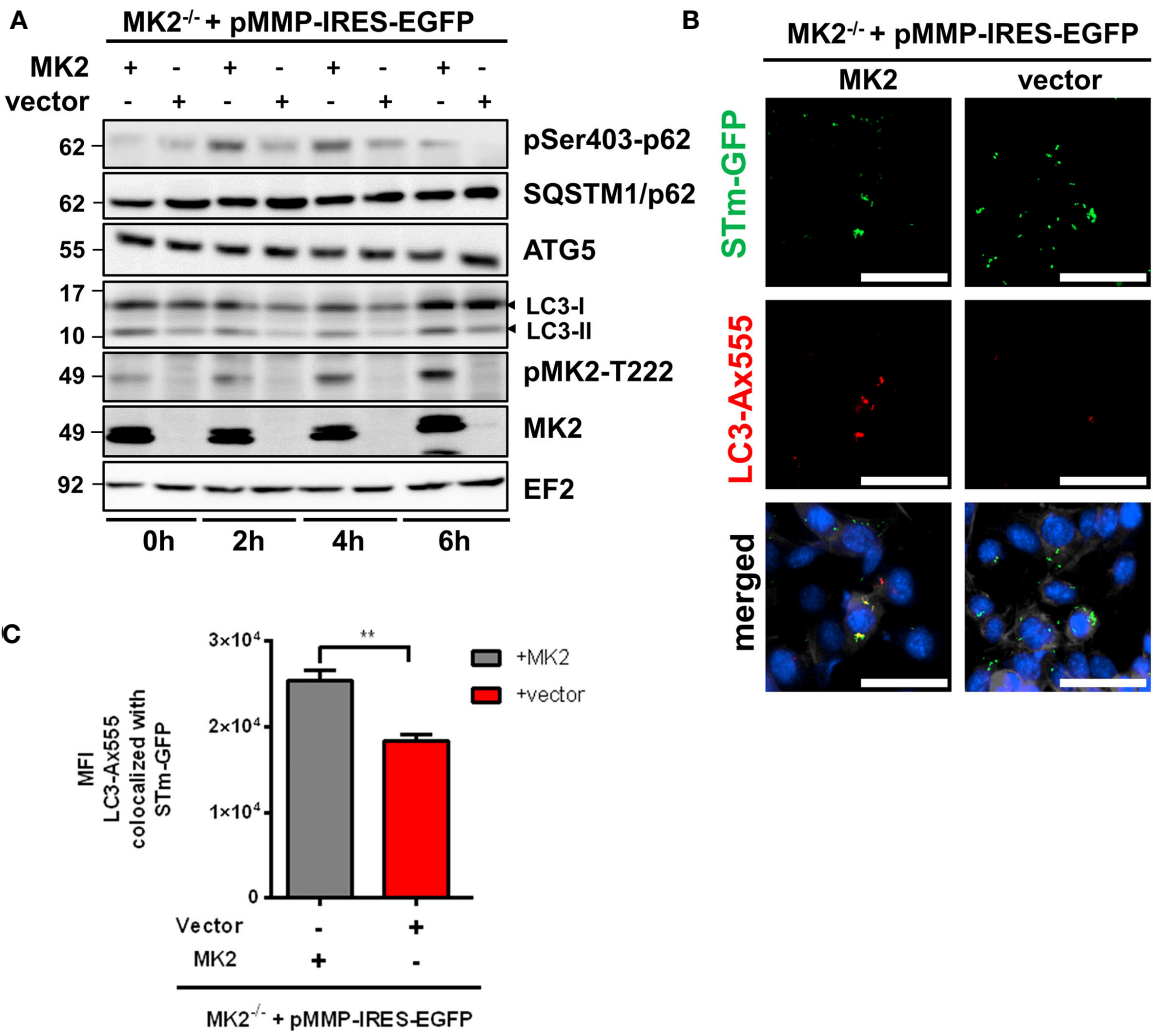
MK2 is required to activate and recruit the autophagy machinery to *S.* Typhimurium. **(A)** *Mk2*-rescued (MK2) and *Mk2^-/-^* (vector) were infected with STm WT at MOI 100. Western blot was performed to cells infected at 0, 2, 4 and 6h p.i,. Representative blots from three independent experiments are shown. **(B)** *Mk2*-rescued (MK2) and *Mk2^-/-^* (vector) were infected with STm-GFP (green) at MOI 100. Infected cells were fixed at 2h p.i, stained with LC3 antibody followed by Ax555-labeled secondary antibody (red). Nuclei were stained with DAPI (blue). F-actin (white) were stained with Phalloidin-647. Original magnification: 200x. Scale bars = 100 μm. **(C)** Quantification of LC3-Ax555 colocalized with STm-GFP. The results are defined as the median of Ax555 fluorescence intensity colocalized with STm-GFP. Unpaired t-test. **p < 0.01.

Autophagy, known for the degradation of cytosolic components in eukaryotic cells, plays an important role in recognizing and protecting cells from invading intracellular pathogens such as *Salmonella*. To understand the mechanisms of MK2 mediated control of susceptibility to *Salmonella* infection, we compared the levels of lipidated LC3 (LC3-II) levels between MK2-deficient and expressing MEFs infected with S. Typhimurium. We found that LC3-II levels after infection were lower in *Mk2^-/-^* MEFs compared to *Mk2*-rescued MEFs (**Figure 3A**). Non-lipidated LC3 (LC3-I) was also upregulated upon infection in both cell lines, with the MK2-rescued MEFs showing slightly increased levels. Mechanisms involved in this upregulation are unknown, but MAPK pathways have been previously reported to regulate LC3-I levels (23). p62 (also known as SQSTM1) is an autophagy receptor and substrate with multiple protein-protein interaction domains, including an ubiquitin-associated (UBA) domain for ubiquitinated cargo binding and an LC3 interaction region (LIR) for binding to LC3. Similar amount of SQSTM1/p62 were detected in the presence or absence of MK2 (**Figure 3A**). Interestingly, p62 phosphorylation at Ser403 was increased in *Mk2*-rescued MEFs at 2 and 4 h p.i and decreased again at 6 h p.i., even when compared to uninfected MEFs. In contrast, *Mk2^-/-^* MEFs showed lower p62 phosphorylation at Ser403 compared to *Mk2*-rescued MEFs at all-time points. In addition, no differences were detected in the levels of ATG5, a protein essential for the early steps in autophagosome formation (**Figure 3A**).

**Figure 4:**
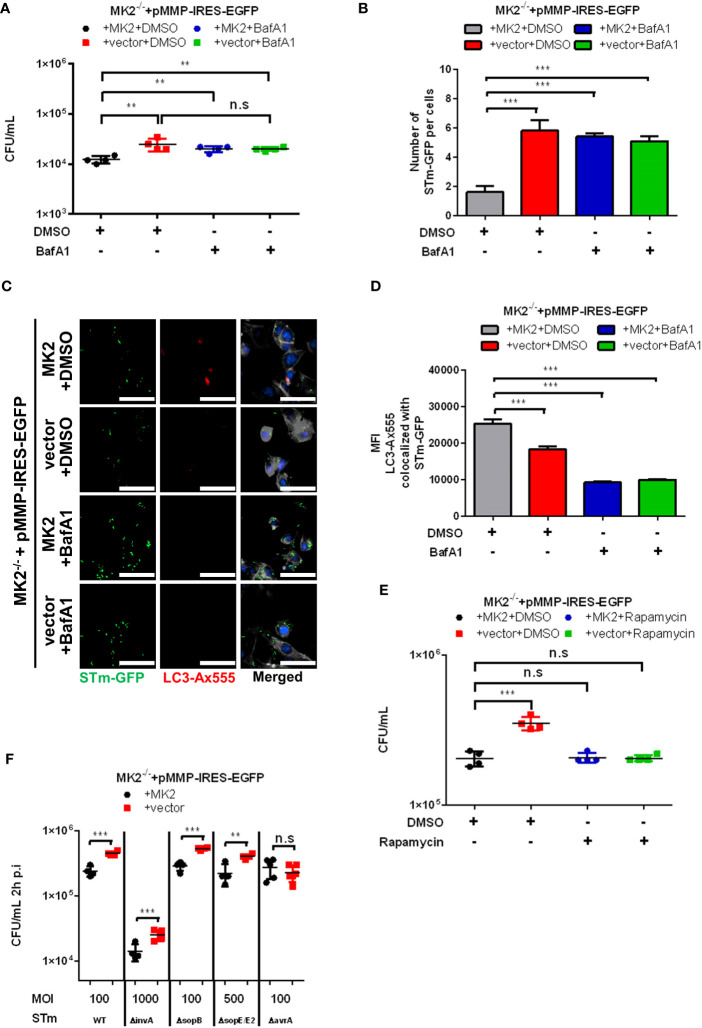
Autophagy activity is required to control intracellular *S.* Typhimurium. **(A)** *Mk2*-rescued (MK2) and *Mk2^-/-^* (vector) treated with DMSO or Bafilomycin A1 (BafA1) were infected with STm WT at MOI 10 for 1 hour, then washed with PBS. Medium with gentamicin (100 μg/mL) and with or without BafA1 were added. At 2h p.i, cells were washed with PBS and lysed. The results are defined as CFU/mL. One-way ANOVA with Tukey’s multiple comparison test, **p<0.01, n.s, not significant. The experiment shown is representative of three independent experiments. **(B)** Quantification and **(C)** immunofluorescence staining of *Mk2*-rescued (MK2) and *Mk2^-/-^* (vector) treated with DMSO or BafA1 were infected with STm-GFP (green) at MOI 100 for 1 hour. Infected cells were fixed at 2h p.i, stained with LC3 antibody followed by Ax568-labeled secondary antibody (red). Nuclei were stained with DAPI (blue). F-actin (white) were stained with Phalloidin-647. The results are defined as number of STm-GFP per cells. Original magnification: 200x. Scale bars = 100 μm. One-way ANOVA with Tukey’s multiple comparison test, ***p < 0.001. **(D)** Quantification of LC3-Ax555 colocalized with STm-GFP. The results are defined as the median of Ax555 fluorescence intensity colocalized with STm-GFP. One-way ANOVA with Tukey’s multiple comparison test, ***p < 0.001. The experiment shown is representative of three independent experiments. **(E)** *Mk2*-rescued (MK2) and *Mk2^-/-^* (vector) with DMSO or rapamycin were infected with STm WT at MOI 10 for 1 hour. Medium with gentamicin (100 μg/mL) and with or without rapamycin were added. At 2h p.i, cells were washed with PBS and lysed. The results are defined as CFU/mL. One-way ANOVA with Tukey’s multiple comparison test, n.s = not significant, ***p<0.001. The experiment shown is representative of three independent experiments. **(F)** *Mk2*-rescued (MK2) and *Mk2^-/-^* (vector) were infected with STm WT, STm Δ*invA*, STm Δ*sopB*, STm Δ*sopE/E2* and STm Δ*avrA* with stated MOI for 1 hour. Medium with gentamicin (100 μg/mL) were added. At 2h p.i, cells were washed with PBS and lysed. The results are defined as CFU/mL. Unpaired t-test. N.s, not significant, **p < 0.01, ***p < 0.001. The experiment shown is representative of three independent experiments.

We then used immunofluorescence microscopy to specifically look at autophagic clearance of *Salmonella*. Co-immunofluorescence staining of MEFs infected with *Salmonella* expressing GFP showed reduced colocalization of *Salmonella* with LC3 in *Mk2^-/-^* MEFs in comparison to *Mk2*-rescued MEFs at 2 h p.i. (**Figures 3B, C**). Similar results were observed in the colocalization of *Salmonella* with the autophagy receptor p62/SQSTM1, known to be essential for efficient autophagy of bacteria (**Supplementary Figures 2A, B**). Inhibition of autophagy with bafilomycin A1 (BafA1) led to increased viable *Salmonella* counts in MK2-expressing MEFs, comparable to that in untreated MK2-deficient cells. This indicates that the positive role of MK2 in restricting *Salmonella* is probably via modulation of autophagy (**Figure 4A**). Consistently, there was no further increase in bacterial counts observed upon treating MK2-deficient cells with BafA1. Immunofluorescence analysis of individual cells infected with *S.* Typhimurium expressing GFP showed increased bacterial numbers after BafA1 treatment at 2h p.i. (**Figures 4B, C**). Furthermore, immunofluorescence staining also showed reduced colocalization of *Salmonella* with LC3 puncta in BafA1 treated cells (**Figures 4C, D**). Interestingly, activation of autophagy by treatment with the mTOR inhibitor rapamycin was sufficient to reduce the bacterial counts in MK2^-^deficient MEFs to levels comparable to that in MK2 expressing cells (**Figure 4E**). This clearly supports a model where p38/MK2 plays essential role in activating selective autophagy of bacterial cells.

It is known that *Salmonella* injects its effector proteins into the host cell cytosol by type III secretion system (T3SS) to avoid the autophagy pathway and for remodeling of the SCV for intracellular survival (24). The T3SS-1 translocated effectors play an important role to influence the host’s cellular functions to facilitate *Salmonella* invasion and mediate their persistence in the host cells (7). AvrA is one of the *Salmonella* effectors secreted by the SPI-1 T3SS and is known to inhibit the host c-Jun N-terminal kinase (JNK)/AP-1 and NF-κB signaling pathways (25) and to suppress autophagy by reducing Beclin-1 protein (26). Therefore, we then infected MEFs with *S.* Typhimurium Δ*avrA* and other SPI-1 mutants such as Δ*invA*, Δ*sopB*, Δ*sopE/*Δ*sopE2*. MEFs infected with *S.* Typhimurium Δ*avrA* showed similar bacterial counts in both *Mk2^-/-^* and *Mk2*-rescued MEFs at 2h p.i. (**Figure 4F**) whereas, other SPI-1 mutants showed increased *S.* Typhimurium counts in *Mk2*-deficient MEFs at 2h p.i. compared to *Mk2*-rescued MEFs, similar to the *S.* Typhimurium WT strain infected MEFs (**Figure 4F**). These data support the idea that p38^MAPK^/MK2 signaling modulates the host cell susceptibility to *Salmonella* infection by affecting the autophagy pathways downstream to AvrA.

### 3.4 Exogenous TNF-α suppresses *Salmonella* counts in MK2^-/-^ MEFs

**Figure 5:**
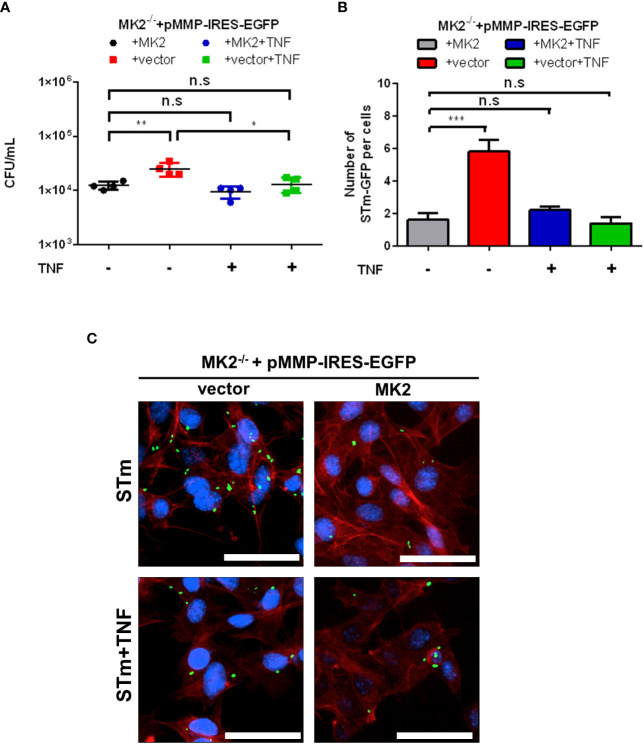
Exogenous TNF treatment influence intracellular *S.* Typhimurium survival in MEFs. **(A)** *Mk2*-rescued (MK2) and *Mk2^-/-^* (vector) treated with or without TNF were infected with STm WT at MOI 10 for 1 hour. Medium with gentamicin (100 μg/mL) and with or without TNF were added. At 2h p.i, cells were washed with PBS and lysed. The results are defined as CFU/mL. One-way ANOVA with Tukey’s multiple comparison test, n.s = not significant, *p<0.05, **p<0.01. The experiment shown is representative of three independent experiments. **(B)** Quantification and **(C)** immunofluorescence staining of *Mk2*-rescued (MK2) and MK2^-/-^ (vector) treated with or without TNF were infected with STm-GFP (green) for 1 hour, fixed at 2h. Nuclei were stained with DAPI (blue). F-actin (red) were stained with Phalloidin-647. Original magnification: 200x. Scale bars = 100 μm. The results are defined as number of STm-GFP per cells. One-way ANOVA with Tukey’s multiple comparison test, n.s = not significant,***p<0.001. The experiment shown is representative of three independent experiments.

It has been known for a long time that MK2 is essential for tumor necrosis factor-α (TNF) biosynthesis (13). Since a previous study also showed that TNF-stimulated p62-mediated autophagic activity restricted survival of *Shigella* and *Listeria* ActA mutant (27), we infected MEFs with *S.* Typhimurium after TNF treatment. Upon treatment with exogenous TNF, we observed decreased *Salmonella* counts in *Mk2^-/-^* MEFs in comparison to untreated *Mk2^-/-^* MEFs (**Figure 5A**). Immunofluorescence analysis in TNF treated *S.* Typhimurium infected *Mk2^-/-^* MEFs showed reduced bacterial number in comparison to untreated MEFs (**Figures 5B, C**). Hence, addition of exogenous TNF can stimulate cellular response, rescues MK2-deficiency and restricts the survival of *S.* Typhimurium.

### 3.5 Reduced TBK1 activation by *Salmonella* infection of *Mk2^-/-^* MEFs

**Figure 6:**
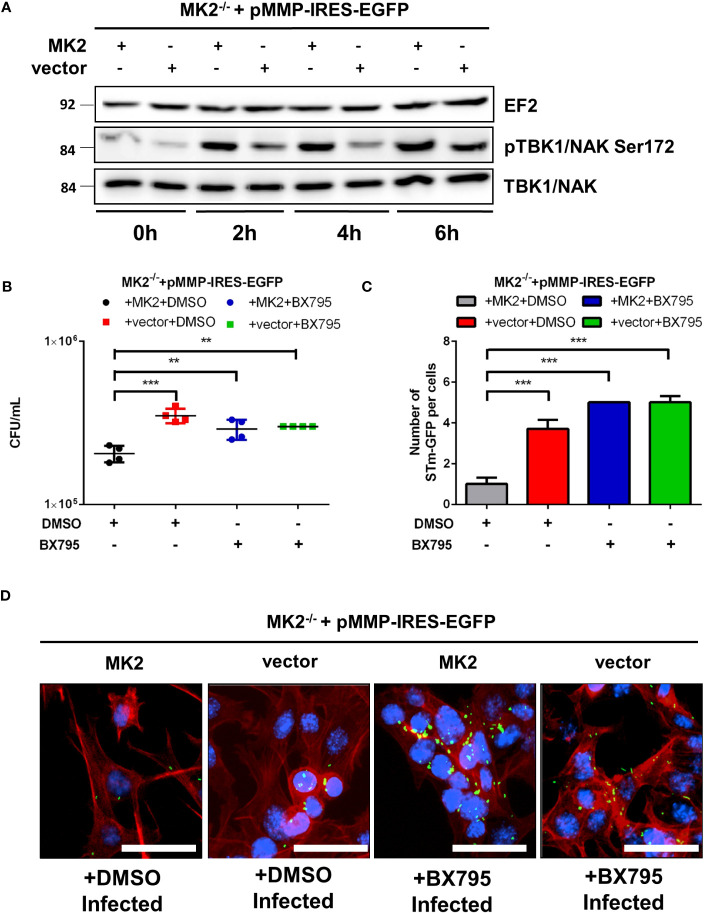
TBK1 activation is required to control intracellular *S.* Typhimurium. **(A)** *Mk2*-rescued (MK2) and *Mk2^-/-^* (vector) were infected with STm WT at MOI 100 for 1 hour. Western blot was performed to cells infected at 0, 2, 4 and 6h p.i,. Representative blots from three independent experiments are shown. **(B)** *Mk2*-rescued (MK2) and MK2^-/-^ (vector) treated with DMSO or BX795 were infected with STm WT at MOI 10 for 1 hour. Medium with gentamicin (100 μg/mL) and BX795 were added. At 2h p.i, cells were washed with PBS and lysed. The results are defined as CFU/mL. One-way ANOVA with Tukey’s multiple comparison test, **p<0.01, ***p<0.001. The experiment shown is representative of three independent experiments. **(C)** Quantification and **(D)** immunofluorescence staining of *Mk2*-rescued (MK2) and *Mk2^-/-^* (vector) treated with DMSO or BX795 for 1 hour were infected with STm-GFP (green), fixed at 2h p.i. Nuclei were stained with DAPI (blue). F-actin (red) were stained with Phalloidin-647. Original magnification: 200x. Scale bars = 100 μm. The results are defined as number of STm-GFP per cells. One-way ANOVA with Tukey’s multiple comparison test, ***p<0.001. The experiment shown is representative of three independent experiments.

TANK-binding kinase-1 (TBK1), an integral component of Type I interferon induction by microbial infection, is both important for autophagic maturation by coordinating assembly and function of the autophagic machinery (28) and protection of vacuolar integrity during intracellular bacterial infection (29). Catalytically active TBK1 is recruited to cytosol-invading *Salmonella* and then induce anti-bacterial autophagy via the upstream autophagy regulator WIPI2 (30) and phosphorylation of the autophagic adaptor p62 at Ser-403 (31). Interestingly, IKKϵ and TBK1 are also activated in cells after stimulation with TNF (32). Since we detected reduced p62-S403 phosphorylation in *Salmonella* infected MK2-deficient cells (**Figure 3A**), we employed western blot to determine whether MK2 signaling affects TBK1 activation. Similar levels of TBK1 were observed in all time points for both genotypes. However, activated TBK1 (pS172-TBK1) was higher in *Mk2*-rescued MEFs after infection in comparison to *Mk2^-/-^* MEFs (**Figure 6A**). In addition, MK2-K79R MEFs showed a level of pS172-TBK1 similar to *Mk2*-rescued MEFs after infection indicating that a rescue of the p38^MAPK^ level by expression of MK2 protein as described in Kotlyarov et al. (33) and Ronkina et al. (19) is sufficient for TBK1 activation (**Supplementary Figure 3**). Furthermore, treatment with the TBK1 inhibitor BX795 resulted in increased *Salmonella* counts in MK2 expressing MEFs (**Figure 6B**) indicating a role for TBK1 in restricting *Salmonella* infection. In agreement with this finding, immunofluorescence microscopy of BX795 treated *S.* Typhimurium infected MEFs showed increased bacterial number in comparison to untreated *S.* Typhimurium infected cells (**Figures 6C, D**). These data indicate that a p38^MAPK^/MK2-dependent activation of TBK1 during bacterial infection is essential to restrict *Salmonella* infection and there is a defect in TBK1-regulated bacterial clearance in *Mk2^-/-^* MEFs.

## 4 Discussion

The p38^MAPK^/MK2 pathway is crucial for innate immunity and the inflammatory response *in vivo* and *in vitro*. In this study, inhibition of p38^MAPK^/MK2 in wild type cells and the use of MK2-deficient cells in a model of early *S.* Typhimurium infection showed increased bacterial survival in MEFs, macrophages and HEK293T cells. In our previous study, MK2-deficient mice displayed higher susceptibility to *L. monocytogenes* infection due to impaired control of bacterial growth. Lower production of TNF and IFN-γ were observed in this infection model *in vivo* and *in vitro*. In addition, decreased phagocytosis of bacteria but normal oxidative burst activity were observed in *Mk2^-/-^* macrophages (14). We also demonstrated previously that p38^MAPK^/MK2 pathway repressed cell apoptosis via RIPK1 in an *Yersinia enterocolitica* infection model (34).

Upon invasion, *Salmonella* used their T3SSs to hijack host factors for their interests including the establishment of SCV and *Salmonella*-induced filaments (SIFs), which are important for their survival inside the host cells. However, some bacteria can penetrate and escape from the SCV and replicate rapidly in the host cell cytosol. From our study, microscopy and chloroquine resistance assays revealed that in the absence of MK2, a higher percentage of bacteria was detected in the host’s cytosol. It is known that upon exposure to the cytoplasm, *Salmonella* are quickly recognized by the ubiquitin E3-ligases of the host cells and ubiquitinated (35). Ubiquitinated bacteria then activate autophagic adaptors such as NDP52, OPTN, and p62, resulting the interaction with autophagic membrane anchoring protein LC3 that lead the bacteria to autophagosomes (27, 36). However, a different mechanism, in which *Salmonella* residing in damaged SCVs could also be targeted for autophagy by galectin 8, further adaptors and ubiquitin resulting in autophagic elimination, has also been described (37).

Autophagy as a part of innate immunity is an important mechanism to eliminate intracellular pathogens. *Salmonella*, *Shigella*, *Listeria*, *Mycobacterium* and other invasive bacterial pathogens have been known to induce autophagy (38). Losier et al. reported that adenosine monophosphate-activated protein kinase (AMPK), an upstream activator or the autophagy pathway, was stimulated upon detection of outer membrane vesicles from *Salmonella*. Upon AMPK activation, mammalian target of rapamycin complex 1 (mTORC1)-mediated repression of autophagy was relieved, positioning the cell for a rapid induction of autophagy against bacteria (39). In our current study, addition of mTOR inhibitor rapamycin showed reduced bacterial counts, which might result from induction of general autophagy. On the contrary, inhibition of autophagy with BafA1 treatment showed increased bacterial counts. Although p38^MAPK^/MK2/MK3 regulated autophagy by phosphorylation of Beclin-1 at Serine 90 upon starvation in HeLa cells has been described (40), it still remains open whether the regulation of autophagy by p38^MAPK^/MK2 upon *Salmonella* infection in MEFs follows the same mechanism.

In this study, we observed that in the absence of MK2, *Salmonella* infection leads to lower levels of LC3-II and serine 403 phosphorylated p62. Likewise, microscopy analysis showed lower colocalization of *Salmonella* with LC3 and p62 in *Mk2^-/-^* cells. During bacterial infection, phosphorylation of Serine 403 in the UBA domain of p62 by TBK-1 increases p62’s affinity for polyubiquitinated proteins and protein aggregates resulting in activation of the autophagic machinery and autophagic clearance (28, 41). Our data indicate that MK2 is upstream to this TBK1-dependent clearance process and that the level of activated TBK1 (pS172-TBK1) was higher in MK2 expressing MEFs after infection in comparison to MK2-deficient MEFs. TBK1 plays important role in many cellular processes such as innate immunity, inflammatory cytokine production, autophagy and cell death. It is activated by pattern-recognition receptors including Toll-like receptors and intracellular receptors including RIG-I, MDA5 or DAI. TBK1 is also important for the activation of type I interferon (IFN) (42). Focal adhesion kinase (FAK)-interacting protein of 200 kDa (FIP200), a component of the autophagy inducing the UNC-51-like autophagy activating kinase 1 (ULK1) complex, controls the TBK1 activation and p62 phosphorylation at Ser403 (31). The initiation of autophagy process is regulated by the ULK1 complex and the class III phosphatidylinositol 3-kinase (PtdIns3K) lipid kinase complex. Beclin 1 (BECN1), the regulatory component of this complex is a substrate of MK2 and a potential mediator of MK2-mediated control of TBK1 activation (40).

During autophagosome induction, TBK1 associates with gradient fractions consisting of the p62 and BECN1-PI3K complex component UVRAG (28). TBK1 activation is a prerequisite for recruitment of cytoplasmic *Salmonella* to autophagosomes (30). *Salmonella* AvrA has been shown to downregulate BECN1 protein levels to suppress autophagy (26). In the context of pathogen-induced autophagy, TBK1 phosphorylation was enhanced in BECN1 knockout cells upon Sendai virus infection, as BECN1 was shown to enhance autophagy and suppress TBK1 activation (43). Therefore, we hypothesize that sensing of *Salmonella* in the cytoplasm leads to TBK1 activation and downstream autophagosome assembly mediated by BECN1 and p62, while the bacteria attempt to inactivate this signaling by degrading BECN1 using AvrA-dependent mechanisms. The lack of MK2-dependence on bacterial survival in AvrA-deficient *Salmonella* indicate that BECN1 could be the target of MK2. TBK1 activation is downregulated and/or delayed in MK2-deficient cells. The complex modification and interactome pattern of BECN1, regulated by MK2-mediated phosphorylation (40) could regulate the balance between autophagy initiation and TBK1 activation. In addition, the MK2-K79R mutant could rescue the TBK1 activation-defect associated with MK2-deficiency. However, the catalytic inactive mutant could not rescue the bacterial susceptibility of MK2 KO cells (**Figure 1F**). It seems that the catalytic activity of MK2 is not necessary to activate TBK1, although its activity is important for eliminating *Salmonella*. Thus, this needs to be studied further and it should be taken into account that expression of both, active and catalytic dead MK2 is able to stabilize p38^MAPK^ and restore p38 signaling (19, 33).

The transcription factor EB (TFEB) regulates various cellular responses including autophagy and the lysosomal pathway in response to different stress conditions, such as nutrient deprivation, organelle damage and pathogen invasion. Recently, it has been demonstrated that p38^MAPK^ phosphorylates TFEB at serine 401, activates numerous immune genes and impairs the polarization of M0 into M1 inflammatory macrophages (44). Also, the localization of TFEB to lysosomes via mTOR interaction results in TFEB phosphorylation at serine 211. Subsequently, phosphorylated TFEB locates to the cytoplasm whereas the dephosphorylated TFEB translocates to the nucleus which then activates its target genes including autophagy and immune genes (45). Interestingly, the small molecule autophagy inducer acacetin can reduce intracellular *Salmonella* infection by inducing TFEB dephosphorylation, thereby increasing autolysosomal and lysosomal populations in the cells and restricting replication of intracellular *Salmonella* (46).

Some pathogens have evolved escape mechanisms to overcome autophagy that are important for their survival inside the cells (9, 38). The various SPI-1 effector proteins identified in *Salmonella* have different roles during *Salmonella* infection, such as alteration of host cytoskeleton, cell metabolism and regulation of host inflammatory response (47). Our current results revealed that SPI-1 mutants such as Δ*invA*, Δ*sopB*, Δ*sopE/*Δ*sopE2* showed increased *S.* Typhimurium counts in *Mk2^-/-^* MEFs that are similar to MEFs cells infected with *S.* Typhimurium WT. The *S.* Typhimurium *invA* is one of the invasion genes required for invasion of non-phagocytic cells (48). The *Salmonella* outer proteins (Sop)B, an inositol phosphatase that is also known as SigD, blocks inositol phosphate signaling pathways and induces Akt activation that inhibit apoptosis (49, 50). SopE and its homolog SopE2, a Rho GTPase exchange factor, activates Cdc42 and Rac1 that induces actin cytoskeleton rearrangements, membrane ruffling and promotes bacterial invasion (51, 52). Obviously, these genes play MK2-independent roles in *Salmonella* infection. Interestingly, only *S.* Typhimurium Δ*avrA* showed similar bacterial counts in *Mk2*-rescued and *Mk2^-/-^* MEFs at 2h p.i. The AvrA protein plays a critical role in suppressing activation of the NF-κB pathway, apoptosis via JNK pathway and autophagy by reducing Beclin-1 protein (26, 53, 54). These functions of AvrA overlap with MK2/3 functions and might explain the phenotype observed in our study.

Our experiments demonstrated that the addition of exogenous TNF showed reduced bacterial numbers in *Mk2^-/-^* MEFs in comparison with untreated *Salmonella* infected *Mk2^-/-^* MEFs. Since it is known that MK2 regulated biosynthesis of TNF and other cytokines at the post-transcriptional level and inactivation the mRNA-destabilizing and translation-inhibiting protein tristetraprolin (TTP) (13, 55), it is likely that addition of TNF could at least partially rescue early effects of MK2/3-deficiency. TNF has been shown to induce autophagy to eliminate intracellular pathogens, such as *Toxoplasma gondii*, *Listeria* and *Shigella* (27, 56). In *Shigella* infection, addition of exogenous TNF increased septin cage formation and entrapment of bacteria to be targeted by autophagy leading to inhibition of *Shigella’s* cell-to-cell spread (57).

However, the precise role of autophagy, TFEB regulation and TNF mediated mechanisms of host defense to limit bacterial dissemination still needs further investigation. In summary, we demonstrated here that p38^MAPK^/MK2 signaling is important to defend cells against intracellular *Salmonella* infection by affecting the autophagic pathways (**Supplementary Figure 4**). Especially, TBK activation seems the critical factor influenced by p38^MAPK^/MK2 signaling upon *Salmonella* infection.

## Data availability statement

The raw data supporting the conclusions of this article will be made available by the authors, without undue reservation.

## Ethics statement

Ethical approval was not required for the studies on humans in accordance with the local legislation and institutional requirements because only commercially available established cell lines were used. Ethical approval was not required for the studies on animals in accordance with the local legislation and institutional requirements because only commercially available established cell lines were used.

## Author contributions

AS, GG, and AK conceived and designed the experiments. AS performed the experiments and analyzed the data. AS, MBM, GG, AK, and MG wrote and edited the paper. All authors contributed to the article and approved the submitted version.

## Supplementary material

Supplementary Figure 1
**(A)** MEFs *Mk2*-rescued (closed circle), *Mk2^-/-^* (closed square) were infected with *S.* Typhimurium WT at MOI 10 for 10 minutes or 30 minutes, washed three times with PBS, then incubated for 1 hours in media supplemented with 100 μg/mL gentamicin. At 70 minutes and 90 minutes p.i, the cells were lysed, collected, serially diluted and plated on LB agar plates to quantify intracellular bacteria. The results are defined as CFU/mL. Unpaired t-test, n.s = not significant. **(B)** iBMDM *Mk2*-rescued and *Mk2^-/-^* (vector) and **(C)** BMDM *Mk^-+/+^* (closed circle), *Mk2^-/-^* (closed square) were infected with *S.* Typhimurium WT at MOI 10 for 30 minutes, washed three times with PBS, then incubated for 1 hours in media supplemented with 100 μg/mL gentamicin. At 1.5h and 4h p.i, the cells were lysed, collected, serially diluted and plated on LB agar plates to quantify intracellular bacteria. The results are defined as CFU/mL. Unpaired t-test, n.s = not significant, **p<0.01. **(D)** HEK293T cells WT (closed circle) and MK2^-/-^ (closed square) were infected with *S.* Typhimurium WT at MOI 10 for 1 hour, washed three times with PBS, then incubated for 1 hours in media supplemented with 100 μg/mL gentamicin. At 2h and 6h p.i, the cells were lysed, collected, serially diluted and plated on LB agar plates to quantify intracellular bacteria. The results are defined as CFU/mL. Unpaired t-test, **p<0.01.

Supplementary Figure 2
**(A)** *Mk2*-rescued (MK2) and *Mk2^-/-^* (vector) were infected with STm-GFP (green) at MOI 100. Infected cells were fixed at 2h p.i, stained with p62 antibody followed by Ax568-labeled secondary antibody (red). Nuclei were stained with DAPI (blue). F-actin (white) were stained with Phalloidin-647. Original magnification: 200x. Scale bars = 100 μm. **(B)** Quantification of LC3-Ax568 colocalized with STm-GFP. The results are defined as the median of Ax568 fluorescence intensity colocalized with STm-GFP. Unpaired t-test. ***p < 0.001.

Supplementary Figure 3 MEFs expressing a kinase-dead MK2 mutant, in which lysine 79 was replaced by an arginine (*Mk2^K79R^*), were infected with STm WT at MOI 100 for 1 hour. Lysates from cells infected for 0, 2, 4 and 6h were separated by SDS-PAGE and TBK1/NAK expression and phosphorylation at S172 (TBK1/NAK Ser 172) were detected by western blot. Elongation factor 2 (EF2) serves as loading control.

Supplementary Figure 4 A proposed model of regulation of intracellular S. Typhimurium (STm) survival by p38MAPK/MK2-dependent modulation of autophagy pathways in the host cells. (1) After entry into the host cells, Salmonella escape from SCV into the host cytosol. (2) p38MAPK/MK2 are activated by cellular stressors, such as inflammatory cytokines (e.g. IL-1 and TNF), oxidative stress, starvation or infection, and regulate the abundance of cytosolic Salmonella after infection. (3) TBK1 is activated and phosphorylates p62 at serine 403 in the UBA domain. (4) UBA domain phosphorylation enhances the activity of p62 and binds to ubiquitinated Salmonella that escaped to the cytosol. (5) In parallel, MK2 phosphorylates Beclin 1 that induces (6) phagophore nucleation, (7) autophagosome formation and (8) fusion of autophagosomes with lysosomes leads to degradation of the bacteria in autolysosomes. (9) AvrA Salmonella effector suppresses autophagy by inhibiting JNK signaling and reducing Beclin-1 protein level. P -phosphorylation; Ub – ubiquitinylation.
