# Supplementary figures and images for: p38MAPK/MK2 signaling stimulates host cells autophagy pathways to restrict Salmonella infection
Source: Front Immunol. 2023 Sep 12;14:1245443. doi: 10.3389/fimmu.2023.1245443 (PMC10523304; doi:10.3389/fimmu.2023.1245443)

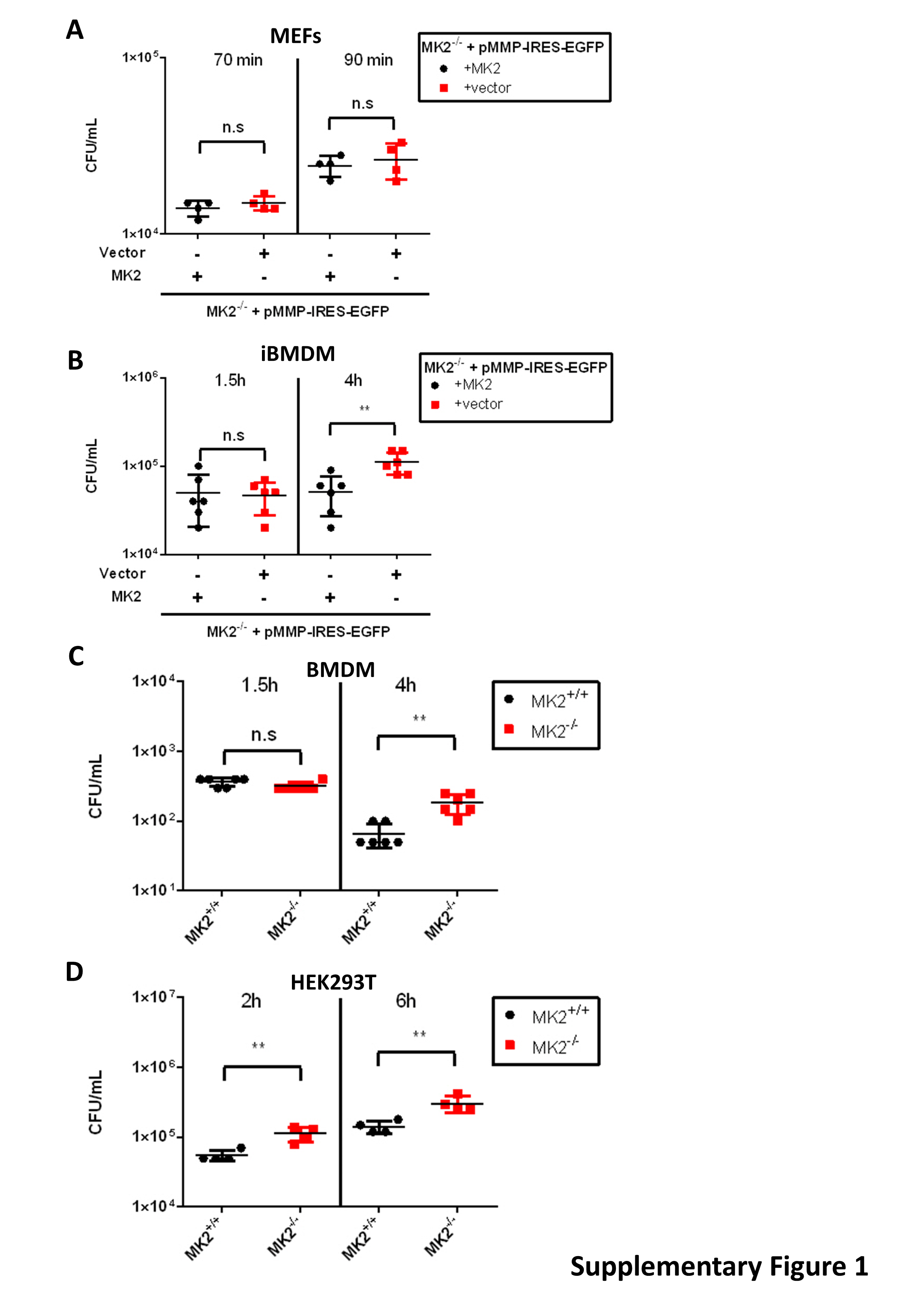

Supplement: Supplementary Figure 1 — (A) MEFs Mk2-rescued (closed circle), Mk2-/- (closed square) were infected with S. Typhimurium WT at MOI 10 for 10 minutes or 30 minutes, washed three times with PBS, then incubated for 1 hours in media supplemented with 100 μg/mL gentamicin. At 70 minutes and 90 minutes p.i, the cells were lysed, collected, serially diluted and plated on LB agar plates to quantify intracellular bacteria. The results are defined as CFU/mL. Unpaired t-test, n.s = not significant. (B) iBMDM Mk2-rescued and Mk2-/- (vector) and (C) BMDM Mk-+/+ (closed circle), Mk2-/- (closed square) were infected with S. Typhimurium WT at MOI 10 for 30 minutes, washed three times with PBS, then incubated for 1 hours in media supplemented with 100 μg/mL gentamicin. At 1.5h and 4h p.i, the cells were lysed, collected, serially diluted and plated on LB agar plates to quantify intracellular bacteria. The results are defined as CFU/mL. Unpaired t-test, n.s = not significant, **p<0.01. (D) HEK293T cells WT (closed circle) and MK2-/- (closed square) were infected with S. Typhimurium WT at MOI 10 for 1 hour, washed three times with PBS, then incubated for 1 hours in media supplemented with 100 μg/mL gentamicin. At 2h and 6h p.i, the cells were lysed, collected, serially diluted and plated on LB agar plates to quantify intracellular bacteria. The results are defined as CFU/mL. Unpaired t-test, **p<0.01. [file Image_1.tif]

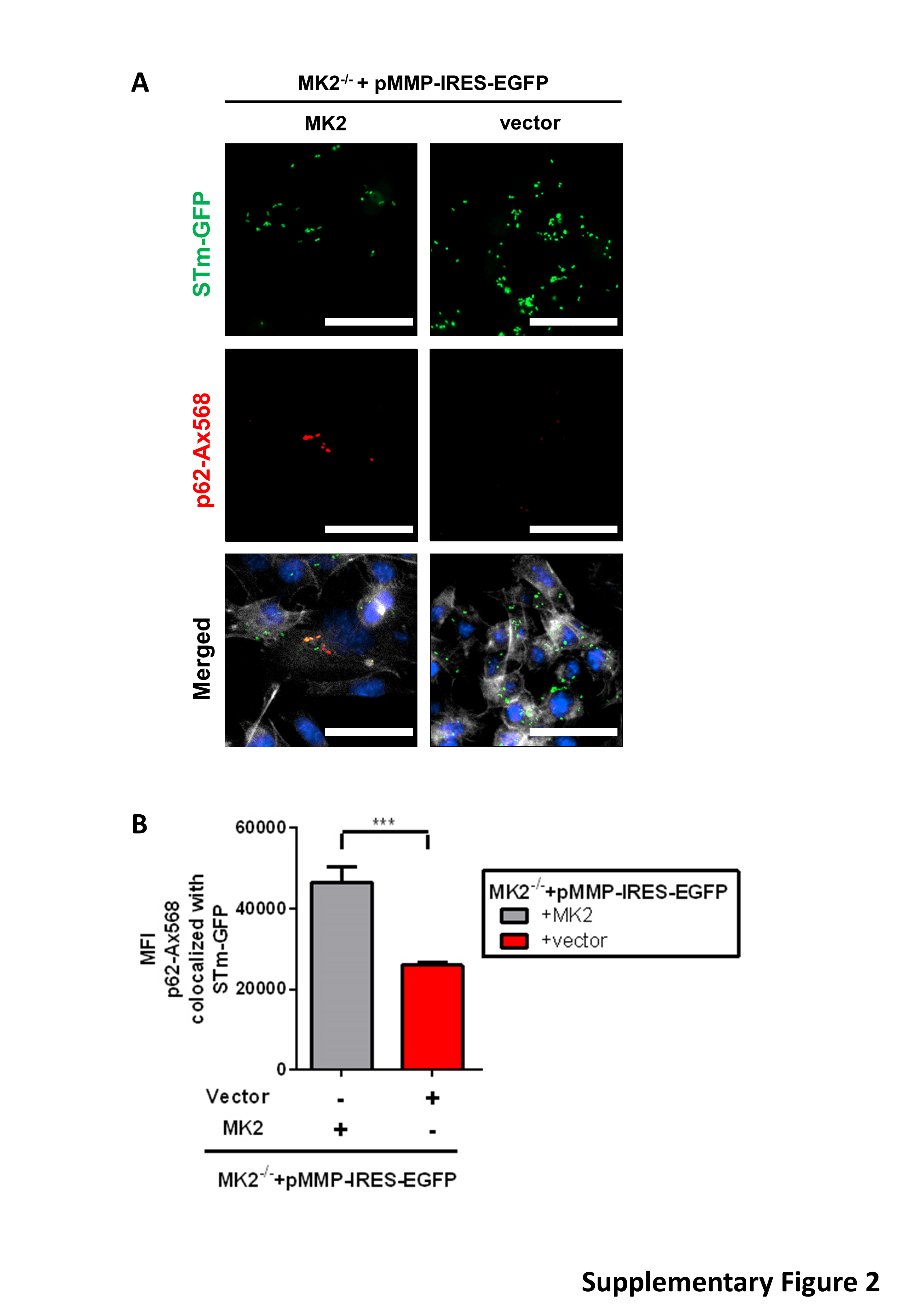

Supplement: Supplementary Figure 2 — (A) Mk2-rescued (MK2) and Mk2-/- (vector) were infected with STm-GFP (green) at MOI 100. Infected cells were fixed at 2h p.i, stained with p62 antibody followed by Ax568-labeled secondary antibody (red). Nuclei were stained with DAPI (blue). F-actin (white) were stained with Phalloidin-647. Original magnification: 200x. Scale bars = 100 μm. (B) Quantification of LC3-Ax568 colocalized with STm-GFP. The results are defined as the median of Ax568 fluorescence intensity colocalized with STm-GFP. Unpaired t-test. ***p < 0.001. [file Image_2.tif]

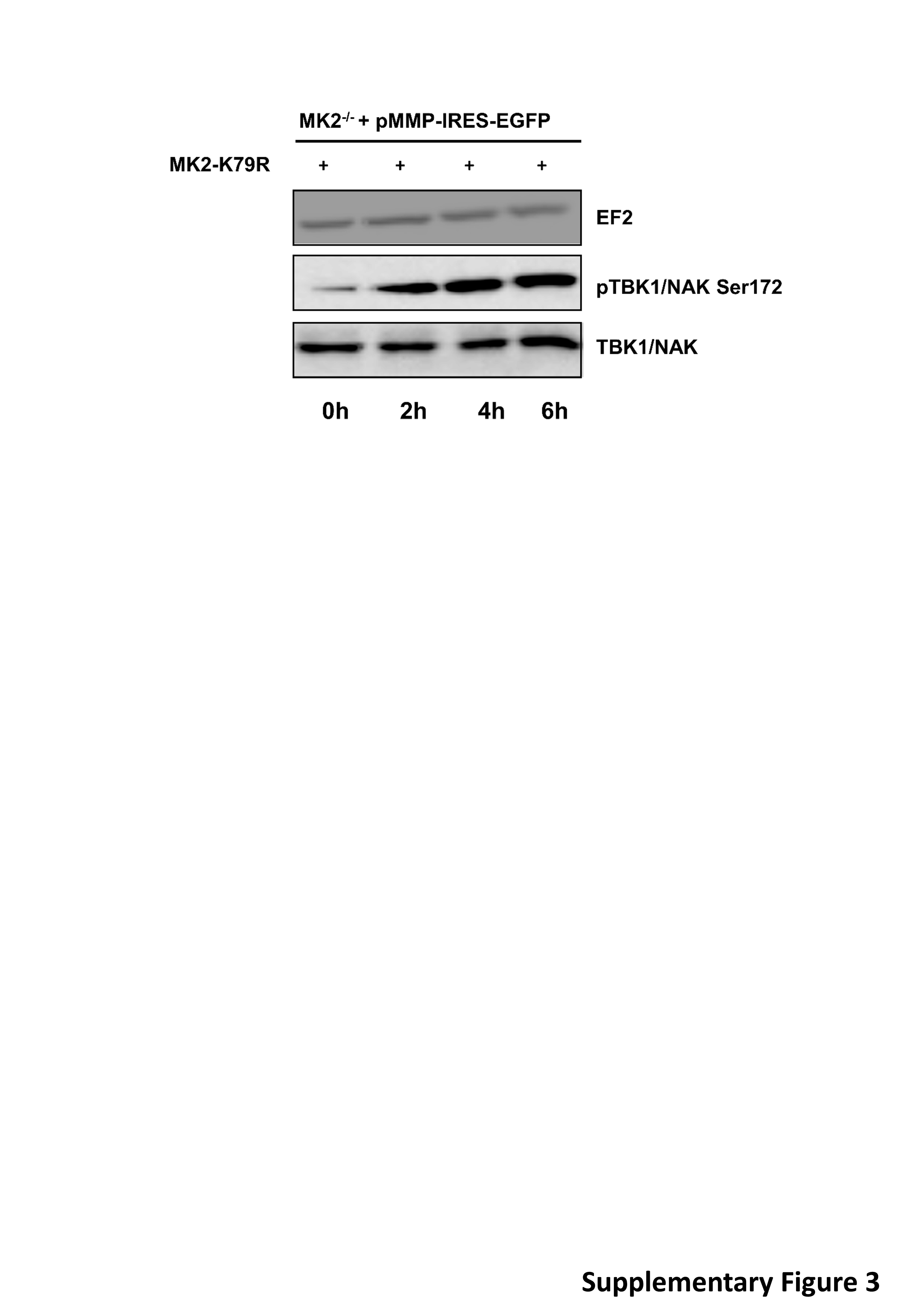

Supplement: Supplementary Figure 3 — MEFs expressing a kinase-dead MK2 mutant, in which lysine 79 was replaced by an arginine (Mk2K79R ), were infected with STm WT at MOI 100 for 1 hour. Lysates from cells infected for 0, 2, 4 and 6h were separated by SDS-PAGE and TBK1/NAK expression and phosphorylation at S172 (TBK1/NAK Ser 172) were detected by western blot. Elongation factor 2 (EF2) serves as loading control. [file Image_3.tif]

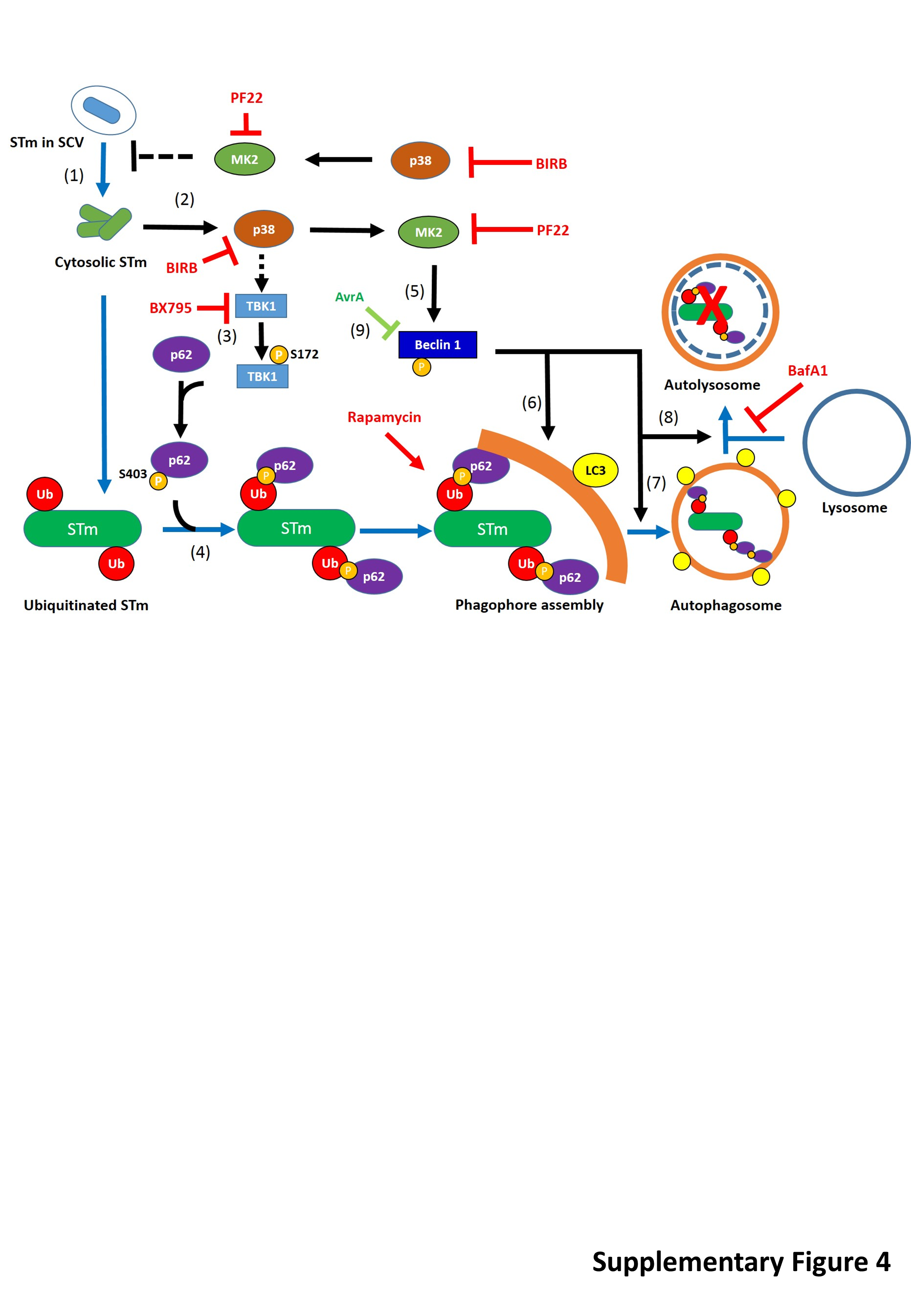

Supplement: Supplementary Figure 4 — A proposed model of regulation of intracellular S. Typhimurium (STm) survival by p38MAPK/MK2-dependent modulation of autophagy pathways in the host cells. (1) After entry into the host cells, Salmonella escape from SCV into the host cytosol. (2) p38MAPK/MK2 are activated by cellular stressors, such as inflammatory cytokines (e.g. IL-1 and TNF), oxidative stress, starvation or infection, and regulate the abundance of cytosolic Salmonella after infection. (3) TBK1 is activated and phosphorylates p62 at serine 403 in the UBA domain. (4) UBA domain phosphorylation enhances the activity of p62 and binds to ubiquitinated Salmonella that escaped to the cytosol. (5) In parallel, MK2 phosphorylates Beclin 1 that induces (6) phagophore nucleation, (7) autophagosome formation and (8) fusion of autophagosomes with lysosomes leads to degradation of the bacteria in autolysosomes. (9) AvrA Salmonella effector suppresses autophagy by inhibiting JNK signaling and reducing Beclin-1 protein level. P -phosphorylation; Ub – ubiquitinylation. [file Image_4.tif]
